# Oral Squamous Cell Carcinoma in Atrophic–Erosive Lichen Planus: 10-Year Rehabilitative Case Report

**DOI:** 10.1155/crid/5548590

**Published:** 2025-02-10

**Authors:** Eduardo Anitua, Laura Piñas, Mohammad H. Alkhraisat

**Affiliations:** ^1^Clinical Research, University Institute for Regenerative Medicine and Oral Implantology (UIRMI), Vitoria, Spain; ^2^Regenerative Medicine, BTI Biotechnology Institute, Vitoria, Spain

## Abstract

Patients with oral lichen planus can sometimes develop malignancy of the process and develop oral squamous cell carcinoma or another type of cancer. Close monitoring of the lesions and early diagnosis is important to increase patient survival. Once cancer treatment has been performed, the therapeutic options for restoring masticatory and phonatory function, in addition to resolving the aesthetic sequelae, are important. In the present clinical case, we show a patient who was treated and followed over a long period of time for both her carcinoma and her subsequent rehabilitation with implants.

## 1. Introduction

Oral lichen planus (OLP) is a chronic inflammatory disease of the mucous membranes, with an estimated prevalence of 2% in the adult population [1–5]. Despite being first described in 1869, its etiology remains under investigation, with autoimmune mechanisms being the most widely studied. Current models suggest that the disease process involves an elevation of tumor necrosis factor-alpha (TNF-*α*), which activates dendritic cells, leading to the recruitment of T lymphocytes. These cells form a characteristic band-like infiltrate along the basal membrane, releasing cytokines such as interleukins (IL) 1, 6, 8, and 10, which play a critical role in the progression of OLP lesions [6–18].

Predisposing factors for OLP include genetic susceptibility, psychological stress, reactions to dental materials, specific medications, and hormonal changes, particularly in women [3, 4, 8–10]. Systemic associations, such as hepatitis C, have also been established, with patients having a significantly higher risk of seropositivity compared to the general population, particularly in regions such as Japan, the Mediterranean, and the United States [13, 14]. Other systemic conditions linked to OLP include diabetes mellitus, autoimmune diseases like lupus erythematosus and Sjögren's syndrome, hypertension, and thyroid disorders. Viral infections, including human papillomavirus (HPV), have been proposed as potential contributors, although evidence remains inconclusive [3, 4, 8–10, 15–40].

The WHO recognizes OLP as an oral potentially malignant disorder [40] due to its potential for malignant transformation. Unlike localized premalignant lesions, OLP represents a systemic inflammatory disease that can create an environment conducive to malignant progression across the entire mucosa [17–50]. The risk of malignancy in OLP is estimated to range from 0% to 14%, with variability influenced by diagnostic criteria and patient factors [51, 52]. Among the clinical subtypes, the atrophic and erosive forms are of particular concern due to their higher association with oral squamous cell carcinoma (OSCC), especially in lingual locations [1–7, 51, 52].

In this report, we present the case of a patient with a history of OLP, initially diagnosed as an erosive form, which subsequently progressed to OSCC. The case includes a detailed description of the diagnostic process, surgical management, and rehabilitation using dental implants, along with a 10-year follow-up. This case highlights the importance of long-term monitoring and the need for individualized therapeutic strategies in high-risk OLP patients.

## 2. Case Presentation

A 67-year-old female patient attended the clinic due to a discomfort, swollen gingiva, and painful sensation in the third quadrant. The patient had been diagnosed with OLP a year earlier, based on clinical findings and an oral biopsy. OLP of atrophic–erosive variant was diagnosed and had required treatment with topical corticosteroids (master formula of triamcinolone acetonide in 0.1% aqueous solution, rinse twice daily for 30 days). As the main systemic pathology of interest, we highlight the presence of Crohn's disease for which he was receiving treatment with infliximab (Samsung Bioepis NL B.V., 2616 LR Delft, Nederland) (1 monthly intravenous dose). She smoked 10 cigarettes per day and was diagnosed with atrophic erosive OLP 1 year ago. Extra orally, no associated lesions were observed at this time or at any time during the course of her lichen planus. The patient completed her treatment with the mouth rinse and returned to the clinic after finishing it (30 days). At this time, a new examination is being conducted as the patient reports having worsened.

**Figure 1:**
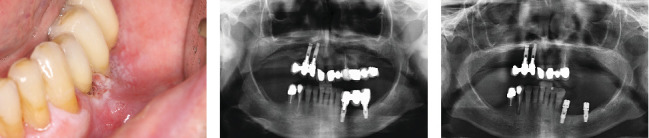
(a) Aspect of the lesion consulted by the patient. We observe the whitish outgrowth areas and how it is introduced under the prosthesis, so we proceed to lift the crowns. (b) X-ray 6 months before the debut of the lesion. (c) Panoramic x-ray on removal of the prosthesis showing bone loss, which may initially suggest peri-implantitis.

On exploration, the patient had a cemented implant-supported partial prosthesis in the third quadrant. An excrescent lesion which followed the gingival contour with whitish areas and a tendency to bleed was observed in the same quadrant (Figure 1(a)). The prosthesis was removed to obtain a biopsy for histopathological analysis, while the implants were left with healing abutments. Once the healing abutments were removed, an x-ray revealed bone loss around both implants, which, along with the mucosal lesion, could initially be mistaken for peri-implantitis. At this point, the mesial bone loss for the implant in Position 34 was measured at 3.94 mm, while the distal bone loss for the same implant was 3.69 mm. For the implant in Position 36, the mesial bone loss was 3.25 mm and the distal bone loss was 1.91 mm. In a previous x-ray taken 6 months prior to the onset of the lesion, the mesial bone loss for the implant in Position 34 was recorded at 0.88 and 1.05 mm distally, while for the implant in Position 36, the mesial bone loss was only 1.59 mm and the distal bone loss was 1.18 mm (Figures 1(b) and 1(c)).

**Figure 2:**
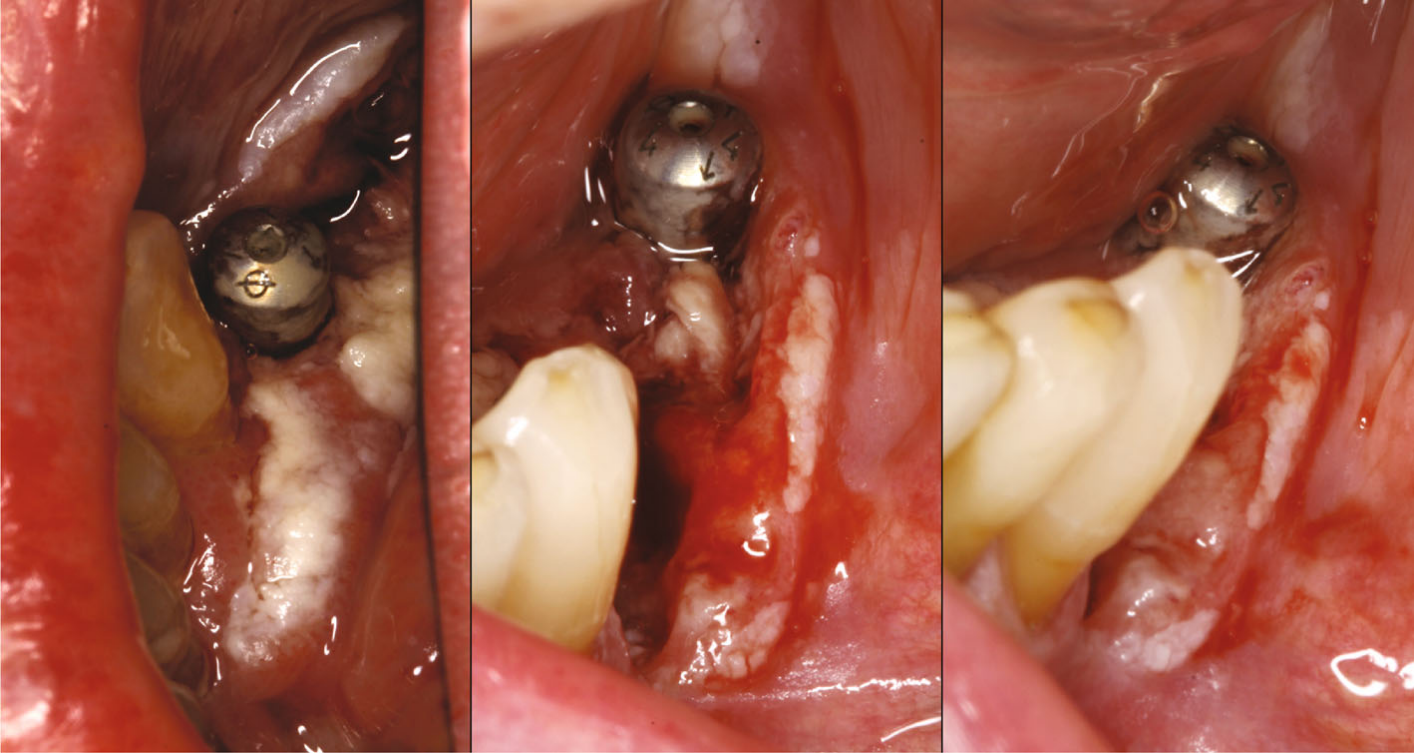
Image of the lesion 20 days after waiting for the histological examination results, confirming the presence of oral squamous cell carcinoma. Lateral view shows the lesion extending along the gingival margin.

**Figure 3:**
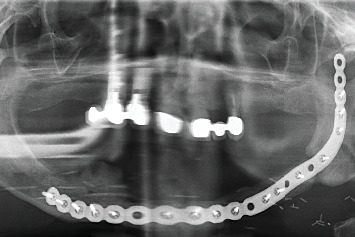
X-ray of the patient with the osteosynthesis plate at the end of the surgery (6 months after resection surgery).

**Figure 4:**
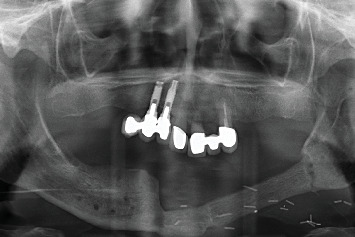
X-ray with bone consolidation and after removal of the osteosynthesis plate (*2.5 years after surgery*).

**Figure 5:**
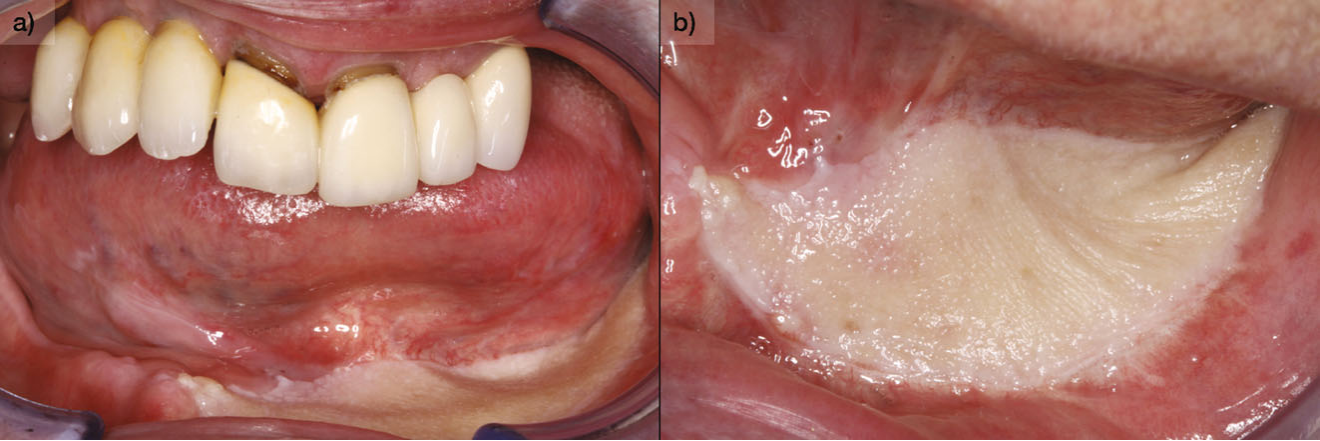
(a) Intraoral images of the patient after surgery with the graft consolidated and healed (6 months after resection surgery). (b) Appearance of the consolidated skin graft on the oral mucosa at higher magnification.

The histopathological results were received in 20 days, confirming a diagnosis of OSCC. During this period, the patient's condition deteriorated significantly; the lesion progressed to an irregular ulcer with poorly defined borders and areas of erythroplasia (Figure 2). The patient was referred to her primary hospital for surgical intervention. Tumor treatment was performed by partial left mandibular resection. The reconstruction of the mandible was achieved by microvascularized peroneal flap and fixation with an osteosynthesis plate (Figure 3). Six months after the surgery, the skin graft was integrated into the oral mucosa (Figures 4 and 5). The bone graft was consolidated into the mandible; the osteosynthesis plate was removed (Figure 3). These implants were placed using low-speed drilling (biological drilling) following the protocol described by Anitua et al., to ensure maximum preservation of the recipient site [53]. To continue with the oral rehabilitation, implant-supported prosthesis was designed to avoid any trauma (pressure or friction) to the underlying tissues. Three implants were positioned (in Positions 43#, 44#, and 46#) at the residual alveolar bone in the fourth quadrant.

**Figure 6:**
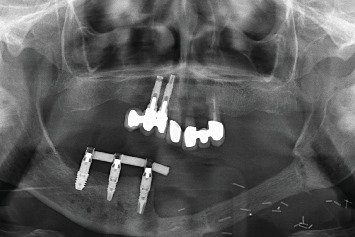
X-ray after placement of the implants and the immediately loaded provisional prosthesis (2.5 years before surgery).

**Figure 7:**
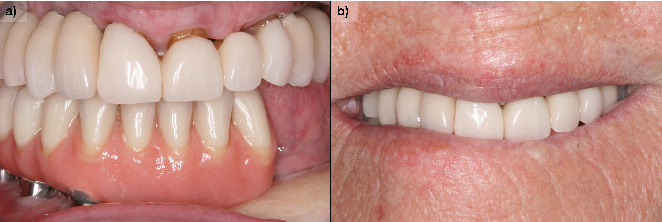
(a) Definitive prosthesis placed in the patient, after 6 months of progressive loading with the initial immediately loaded prosthesis. (b) Image of a smile with the definitive prosthesis.

A partial prosthesis with mesial cantilever into the third quadrant was delivered (Figure 6). The prosthesis was screw-retained to facilitate the follow-up of the lesion and the maintenance of the implants. In this case, the cantilever is slightly extended toward the third quadrant, not considering it appropriate to place implants in the intervened bone, since neither the vascularization of the bone nor the biomechanical behavior that the load of the implants could exert at this level is known. Two more implants are inserted in the upper jaw (there were already two in Positions 11# and 12#) to achieve complete rehabilitation of the quadrant, in Positions 15# and 16#. Two implant-supported prostheses were placed in the maxilla to restore bilateral partial edentulism (Figure 7).

**Figure 8:**
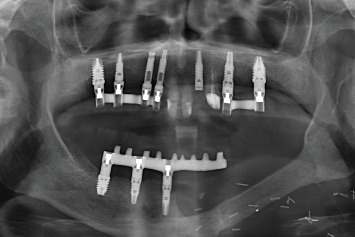
Panoramic radiograph after placement of the definitive prosthesis with the extension to the third quadrant (3 years after surgical resection).

**Figure 9:**
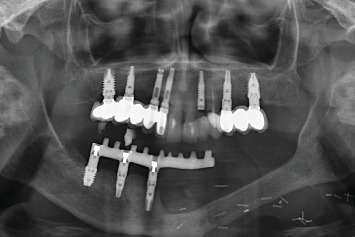
Implant placed in Position 22 to subsequently extract the remaining teeth and make a complete upper prosthesis with the implants already placed previously. Five years have now passed since the surgery to treat oral squamous cell carcinoma.

**Figure 10:**
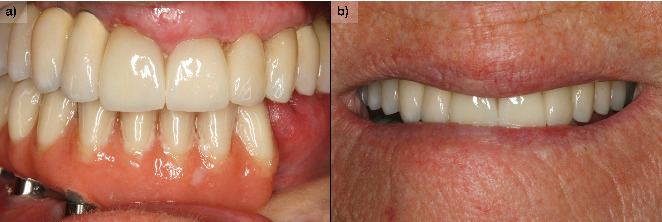
(a) Patient at 10 years of follow-up with definitive upper and lower prostheses. (b) Image of the patient's smile at the end of the follow-up.

**Figure 11:**
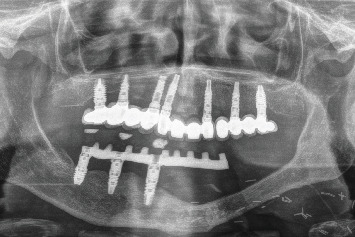
X-ray at 10 years of follow-up with the stability of both the upper and lower implants, as well as the bone grafted in the mandible as can be seen where it is no longer possible to differentiate the points of union to the original mandible.

During the follow-up, a new implant was placed at the position of the second left lateral incisor; the remaining teeth (two central incisors and left upper canine) were extracted due to bone loss, mobility, and pain. Two new prostheses were delivered to restore the edentulous span between the two lateral incisors and the span located distal to the upper left lateral incisor (Figure 8). During the 10 years of follow-up, the patient remained free of new OSCC-associated lesions. The lichen planus was properly controlled by regular check-ups every 3 months and the use of topical corticosteroids (triamcinolone acetonide 0.1% oral solution, rinsed twice a day) in case of new associated erosive or painful lesions. The implants and the prosthesis showed no complications, and none were lost. At the end of follow-up, the bone loss for the implant in Position 43# was 5.27 mm mesially and 4.55 mm distally. For the implant in Position 44#, the mesial bone loss was 0.2 mm and distal 0.40 mm, and for the implant in Position 46#, the mesial bone loss was 0.20 mm and distal 0.30 mm (Figures 9, 10, and 11). Written informed consent was obtained from the patient for the publication of this case report, including the use of personal data and medical history.

## 3. Discussion

OLP is a mucocutaneous and chronic disease very frequent in the dental clinic [1–4]. The dental rehabilitation of patients with OLP has been a challenge in the dental practice, mainly due to their inability to use removable mucosa-supported prostheses and hypersensitivity to certain dental materials routinely used in conventional prostheses [54, 55]. Dental implants in these patients are another therapeutic option to be considered, since different studies have not found a higher rate of failure, bone loss, or incidence of peri-implantitis [56, 57].

The exact mechanism of lichen planus progression to OSCC is still unclear. The most frequently related factors that increase the risk of malignant transformation are hereditary predisposition, tobacco and alcohol consumption, immunosuppressive agents, association with viruses (such as HPV), and the persistence of the chronic inflammatory process [51, 52]. Chronic immunosuppression is a critical factor that can contribute to the malignant transformation of various lesions, including lichen planus. The immune system plays a vital role in surveilling and eliminating potentially malignant cells. When immunosuppression occurs, this surveillance is compromised, allowing for the accumulation of genetic mutations and the progression of premalignant lesions to malignancy. In the context of lichen planus, this could manifest as an increased risk of transformation to OSCC [57–59]. In the presented case, the patient's treatment with anti-TNF-alpha agents for Crohn's disease could indeed represent a significant predisposing factor for the development of OSCC. Anti-TNF-alpha therapy is known to suppress the inflammatory response, which, while beneficial in managing autoimmune conditions, may also lead to reduced immune function.

Additionally, the chronic inflammation associated with Crohn's disease itself may create a tumor-promoting microenvironment that could enhance the likelihood of malignant transformation in coexisting conditions, such as lichen planus. This interplay between chronic immunosuppression, persistent inflammation, and viral infections underscores the complexity of the pathogenesis leading to OSCC in patients with a history of lichen planus.

According to the latest data from a meta-analysis of 20.095 patients with lichen planus and lichenoid reactions, the malignancy rate of OLP is 1.1% [57]. Other studies report malignancy rates ranging from 0% to 14% [51, 52]. There is therefore great controversy as to whether OLP has undergone a process of malignant transformation or whether there are lesions that have not been correctly categorized as lichen planus, presenting a certain tendency to dysplasia from the outset [29].

In this case, our patient did have a previous diagnosis of OLP with confirmed histology without dysplasia, so it is more likely that there was progression from erosive OLP to OSCC. The tumor was developed adjacent to dental implants in the mandible of a female patient. The location and initial manifestation of the OSCC in this case report is in accordance with the conclusions of a recent systematic review [59]. It has been estimated that 88.8% of the OSCC adjacent to dental implants has been in the mandible and the most common elementary lesions have been exophytic growth and ulceration. Most of the patients have been female.

The use of cemented prosthesis would hinder proper examination of the pathological lesion adjacent to dental implants. The removal of the cemented prosthesis has been necessary to obtain properly the biopsy. Thus, the use of screw-retained prosthesis could be recommended to support regular maintenance and proper follow-up of erosive lichen planus lesion adjacent to dental implants. The presence of marginal bone loss may lead to misdiagnosis the lesion as peri-implantitis [59]. The persistence of peri-implantitis around dental implants should be carefully assessed to exclude other pathological lesions. There has been no recurrence of the OSCC during the 10 years of follow-up. In one study, most of OSCC lesions in patients with OLP cases have not relapsed and the disease-free survival rate after 69.8 months has been 97.3% [60].

The oral rehabilitation after partial mandibular resection and reconstruction with microvascularized fibula flap has been performed with implant-supported prosthesis. This type of flap would be the best option when composite tissue reconstruction is required. The outcomes (healing, functional, and aesthetic outcomes) are best obtained by free tissue [61]. For example, nonunion and late complications have been higher in osteocutaneous radial forearm flap than fibula free flap [62]. Most of the complications of microvascularized fibula flap have been in low body mass patients and patients treated with radiotherapy and/or chemotherapy [63, 64]. The healing in this case report has been uneventful.

The dental implants in this case report has been placed in the remaining native mandibular bone to be surrounded by gingival tissues. A cantilever extension has been designed to restore the teeth at the reconstructed region. Although the osseointegration success of the implants placed in the fibula is high (range: 86%–99%), the dental prosthesis success rate is inferior [65]. In our case, the level of the fibula graft has been inferior to the native bone. This discrepancy would increase the crown length and predispose to prosthetic complications [64]. The soft tissue seal around the dental implants would be affected by the tissue quality in the reconstructed area [65]. Indeed, the dental implant in the lower left canine has dermal tissue and has higher marginal bone loss than the other implants surrounded by oral gingival tissue.

This case report has shown the long-term stability of implant-supported prothesis for the oral rehabilitation of erosive lichen planus patient who developed OSCC and reconstructed with microvascularized fibula flap after partial mandibulectomy. This study is limited by its design (case report) and retrospective nature that is dependent of data availability. Further studies are needed to compare the performance of dental implants according to the type of soft tissue (gingival vs. dermal) and to establish recommendations for the clinical practice.

## 4. Conclusions

Screw-retained and implant-supported prosthesis would better support the surveillance and follow-up of potentially malignant lesions such as erosive OLP. The 10-year follow-up data indicated the long-term stability of the implant-supported prosthesis. Gingival tissue around the dental implants would allow for better marginal bone stability. Given the potential for malignant transformation and the chronic nature of OLP, it is crucial to emphasize the importance of thorough and frequent long-term monitoring in these cases to ensure early detection of any changes and to manage the condition effectively.
